# CsEXL3 regulate mechanical harvest-related droopy leaves under the transcriptional activation of CsBES1.2 in tea plant

**DOI:** 10.1093/hr/uhae074

**Published:** 2024-03-07

**Authors:** Haoran Liu, Lingxiao Duan, Jianqiang Ma, Jiqiang Jin, Rong Huang, Yujie Liu, Si Chen, Xiaoying Xu, Jiedan Chen, Mingzhe Yao, Liang Chen

**Affiliations:** Key Laboratory of Biology, Genetics and Breeding of Special Economic Animals and Plants, Ministry of Agriculture and Rural Affairs, Tea Research Institute of the Chinese Academy of Agricultural Sciences, Hangzhou 310008, China; Key Laboratory of Biology, Genetics and Breeding of Special Economic Animals and Plants, Ministry of Agriculture and Rural Affairs, Tea Research Institute of the Chinese Academy of Agricultural Sciences, Hangzhou 310008, China; Key Laboratory of Biology, Genetics and Breeding of Special Economic Animals and Plants, Ministry of Agriculture and Rural Affairs, Tea Research Institute of the Chinese Academy of Agricultural Sciences, Hangzhou 310008, China; Key Laboratory of Biology, Genetics and Breeding of Special Economic Animals and Plants, Ministry of Agriculture and Rural Affairs, Tea Research Institute of the Chinese Academy of Agricultural Sciences, Hangzhou 310008, China; Key Laboratory of Biology, Genetics and Breeding of Special Economic Animals and Plants, Ministry of Agriculture and Rural Affairs, Tea Research Institute of the Chinese Academy of Agricultural Sciences, Hangzhou 310008, China; Key Laboratory of Biology, Genetics and Breeding of Special Economic Animals and Plants, Ministry of Agriculture and Rural Affairs, Tea Research Institute of the Chinese Academy of Agricultural Sciences, Hangzhou 310008, China; Key Laboratory of Biology, Genetics and Breeding of Special Economic Animals and Plants, Ministry of Agriculture and Rural Affairs, Tea Research Institute of the Chinese Academy of Agricultural Sciences, Hangzhou 310008, China; Key Laboratory of Biology, Genetics and Breeding of Special Economic Animals and Plants, Ministry of Agriculture and Rural Affairs, Tea Research Institute of the Chinese Academy of Agricultural Sciences, Hangzhou 310008, China; Key Laboratory of Biology, Genetics and Breeding of Special Economic Animals and Plants, Ministry of Agriculture and Rural Affairs, Tea Research Institute of the Chinese Academy of Agricultural Sciences, Hangzhou 310008, China; Key Laboratory of Biology, Genetics and Breeding of Special Economic Animals and Plants, Ministry of Agriculture and Rural Affairs, Tea Research Institute of the Chinese Academy of Agricultural Sciences, Hangzhou 310008, China; Key Laboratory of Biology, Genetics and Breeding of Special Economic Animals and Plants, Ministry of Agriculture and Rural Affairs, Tea Research Institute of the Chinese Academy of Agricultural Sciences, Hangzhou 310008, China

## Abstract

Due to a labor shortage, the mechanical harvesting of tea plantations has become a focal point. However, mechanical harvest efficiency was hampered by droopy leaves, leading to a high rate of broken tea shoots and leaves. Here, we dissected the genetic structure of leaf droopiness in tea plants using genome-wide association studies (GWAS) on 146 accessions, combined with transcriptome from two accessions with contrasting droopy leaf phenotypes. A set of 16 quantitative trait loci (QTLs) containing 54 SNPs and 34 corresponding candidate genes associated with droopiness were then identified. Among these, CsEXL3 (EXORDIUM-LIKE 3) from Chromosome 1 emerged as a candidate gene. Further investigations revealed that silencing *CsEXL3* in tea plants resulted in weaker vascular cell malformation and brassinosteroid-induced leaf droopiness. Additionally, brassinosteroid signal factor CsBES1.2 was proved to participate in *CsEXL3-*induced droopiness and vascular cell malformation via using the *CsBES1.2*-silencing tea plant. Notably, CsBES1.2 bound on the E-box of *CsEXL3* promoter to transcriptionally activate *CsEXL3* expression as CUT&TAG based ChIP-qPCR and ChIP-seq suggested *in vivo* as well as EMSA and Y1H indicated *in vitro*. Furthermore, CsEXL3 instead of CsBES1.2 decreased lignin content and the expressing levels of lignin biosynthesis genes. Overall, our findings suggest that CsEXL3 regulates droopy leaves, partially through the transcriptional activation of CsBES1.2, with the potential to improve mechanical harvest efficiency in tea plantations.

## Introduction

Tea, crafted from the delicate shoots of the tea plant (*Camellia sinensis* (L.) O. Kuntze), ranks among the world’s favorite non-alcoholic beverages. According to the International Tea Committee, global tea consumption reached to 6.48 million tons in 2022, with China leading the consumption charts at 2.45 million tons. Tea plucking was the key processing method for high economic value from cultivation to market in the tea industry [1]. The low rate of broken leaves was an important goal for tea plucking to achieve as broken leaves impede tea processing and devalue the product [1–3]. Due to the lack of workflow and the high cost of manual harvesting, the tea industry has increasingly shifted its focus towards mechanical harvesting in recent years [2]. Current tea harvest machines could be divided into two types, including intellectual picking machines equipped with visual recognition and manipulators as well as traditional large-scale plucking machines with serrated rotating bits [1, 2, 4]. Despite the potential of intellectual picking machines, their widespread adoption has been limited by challenges such as high costs associated with image recognition in certain environments and the inefficiency of localization in the picking section [1, 4]. Consequently, traditional large-scale plucking machines remain the predominant mechanical method for tea harvesting today [2]. For traditional mechanical plucking on canopy, leaf architectures, including blade angle, droopiness, and blade strength, played a pivotal role in minimizing the rate of broken leaves while directional bits cut nondirective growing tea shoots.

Leaf architecture includes many traits, such as droopiness, blade angle, and leaf area. Droopiness and blade angle are primarily determined by diverse factors, such as proliferation and lignin accumulation of cells inside the midrib [5, 6]. Phytohormones, especially brassinosteroids (BRs), were considered one of the main effectors in modulating these factors and therefore regulating droopiness [5]. BRs are critical for plant vegetative growth and development [7], functioning via a complete signal pathway that extends from cell surface BR receptor to downstream transcription factors, including BES1 (BRI1-EMS-SUPPRESSOR 1) and BZR1 (BRASSINAZOLE-RESISTANT 1). These factors regulate the expression of BR-responsive genes, such as *EXO* (*EXORDIUM*). *EXO* and *EXL* (*EXORDIUM-LIKE*) were homologous genes with the *PHOSPHATE INDUCED-1* gene from tobacco because of their conserved amino acids region (FAM entryPF04674) [8, 9]. EXO/EXL genes were involved in growth, cell expansion, and C starvation stress responses in *Arabidopsis* [8, 10, 11] as well as promoting leaf size in cotton [12]. Overexpressing of *EXO* promoted the expressing levels of *KCS1* and *EXP5* during growth regulation in *Arabidopsis* [10]. Although the participants of BR-related genes in leaf architecture have been studied in different crops, such as *Arabidopsis* or cotton [11, 12], the relationship between *EXO/EXL* and leaf droopiness has been less explored and the functions of EXL, particularly in relation to leaf droopiness, remain to be fully uncovered.

Evidence shows that the BR synthesis enzyme and BR signal pathway components participate in leaf architectures [5, 6]. For instance, *brassinosteroid C-6 oxidase 1* altered BR contents and leaf angle under the transcriptional activation of ZmRAVL1 in maize [6]. In foxtail millet, the DPY1 acts as an interacting regulator with the BR receptor to prevent vascular sclerenchyma malformation and maintain lignin content, and therefore holds leaf erection [5]. Moreover, the BZR1/BES1 family genes have been identified as regulators of leaf bending in rice through antagonistically transcriptional regulation of the *LEAF AND TILLER ANGLE INCREASED CONTROLLER* (*LIC*) [13].

However, few researches focus on candidate genes that regulate the leaf architecture of tea plants, especially for droopiness, because of the difficulty in transgene technology and the long-term for xylophyta hybridization in tea plants. GWAS provided an effective way to identify genes that regulate leaf growth and development from natural tea plant resources or hybrid progeny [14–16]. For example, 25 QTLs that were associated with leaf area and related differentially expressed genes near these QTLs were selected from GWAS of 96 F1 hybrid offspring [15]. Tetratricopeptide repeat (TPR) protein and AT-rich interactive domain protein were significantly associated with leaf serration density and depth according to GWAS based on 120 ancient tea plants, respectively [14]. *CsDREB17* underlying the major QTL of *qTBF4–1* was verified to regulate bud flush of tea plant by conjoint analysis of GWAS and transcriptome [16].

In our study, we first summarized the droopiness among natural tea genetic resources and identified CsEXL3 to be associated with BR-induced leaf droopiness of tea plants based on the integration of GWAS and RNA-seq analysis in natural resources. Then *CsEXL3* was proven to regulate droopiness-related cell expansion at the transcriptional level by using the *CsEXL3-*silencing tea plant. The BR signal pathway component, CsBES1.2, was involved in *CsEXL3-*induced droopiness. The transcriptional regulation of CsBES1.2 on *CsEXL3* was further confirmed *in vitro* and *in vivo,* such as ChIP-qPCR via CUT&TAG. The regulations of *CsEXL3* on lignin content in retarded cells of leaf midrib and the expressing levels of lignin biosynthesis-related genes were also described.

## Results

### Variation in droopy leaves among different germplasms

**Figure 1:**
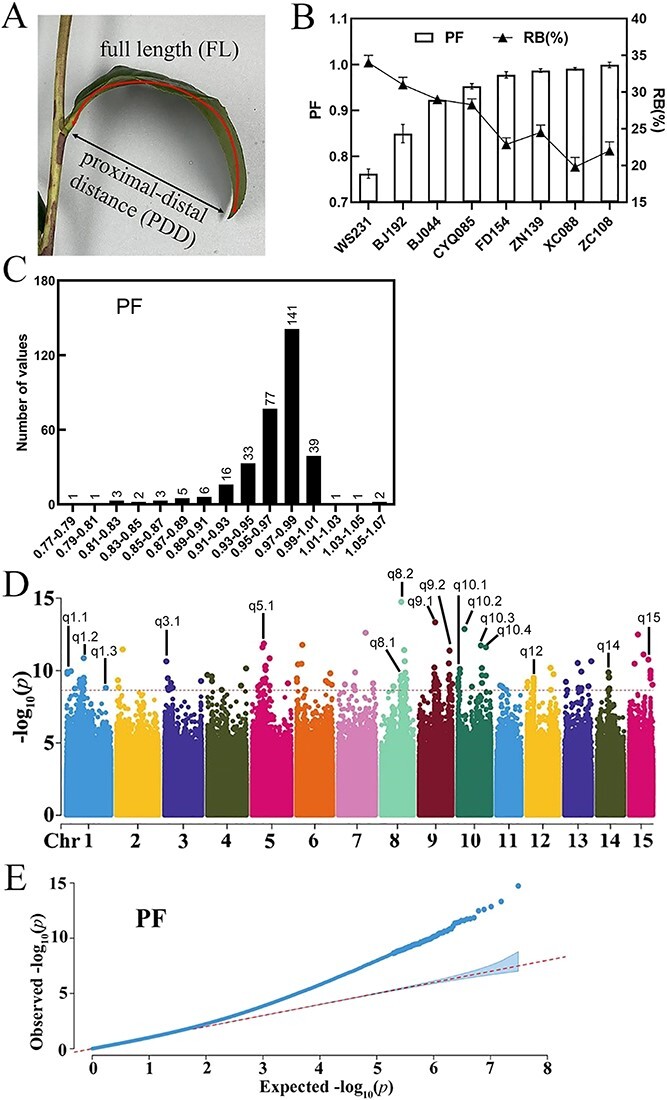
Genome-wide association study of droopy leaves. (**A**) Diagram illustrating the proximal-distal distance (PDD) and full length (FL) of a leaf blade. The ratio (PF) of proximal-distal distance (PDD) to full length (FL) was used to measure curling degree of the leaf blade. (**B**) The relationship between PF and the ratio of broken leaves (RB) after mechanical harvest. (**C**) Variation and distribution of droopiness in tea germplasms. (**D**) Manhattan plot according to genome-wide association study (GWAS) for PF. (**E**) The correlation analysis Q-Q plot according to genome-wide association study for PF.

The ratio of the proximal-distal distance to full length in a leaf blade (PF) was employed to distinguish droopy leaves, as outlined in previous research [5] (Fig. 1A). Droopy leaves exhibited lower PF values as the bending pattern reduced the proximal-distal distance. The PF of straight leaves was close to the value of 1.

To elucidate the correlation between PF and the rate of broken leaves (RB), accessions with higher PF values, such as ‘Zhongcha 108’ (ZC108) and ‘Xicha 5’ (XC088), or lower PF values, such as ‘Wushi’ (WS231) and ‘Jianghongzhong’ (JHZ49), were selected and subjected to machine harvest during spring (Fig. 1B). Droopy leaves with lower PF demonstrated higher RB percentages. For example, the PF mean of WS231 was 0.76 and the RB of WS231 was 34.52%. Conversely, ZC108, which had a PF of 0.99, showed a lower RB of 22.01%.

A high rate of broken leaves after machine harvest hampers further tea processing [1–3]. Identification of droopiness-regulating genes points out the breeding direction of tea plants that are suitable for machine harvest. Leaf droopiness of tea plant is clearly a quantitative trait. The droopiness variation and distribution of selected 331 accessions were summarized according to the value of PF in Fig. 1C. The average of PF was 0.96 ± 0.04 and the value of PF varied from 0.77 to 1.07. The distribution exhibited a kurtosis of 7.41 and a skewness of −2.15 (Table S1, see online supplementary material). After the droopiness investigation of accessions, a genome-wide association study was performed among 146 of these 331 accessions to identify the droopiness-regulating gene.

GWAS for the value of PF using a mixed linear model implemented in the EMMAX program was performed and significant association signals were detected with the threshold (−log_10_(*p*), *P*-value = 2.34E-09) above 8.6 according to the determination of significant candidate loci threshold via GEC software (Fig. 1D and E). SNPs that were located nearby ±1Mbp of the genes coding region (the locations of genes were determined according to ‘Shachazao’ V2, http://tpia.teaplants.cn/) were selected and SNPs from the same region were attributed to one QTL. Then, 16 QTLs containing 54 SNPs were identified across nine chromosomes (Table S2, see online supplementary material). As tea plant is xylophyta with a high degree of genetic heterozygosity, the 340 kb region covered by the above 16 QTLs was determined to delineate candidate genes. Genes with no expressing value or an expressing value below five in four types of tissues, including apical buds, young leaf, mature leaf, and old leaf, were excluded based on reference expressing data (‘Shachazao’ 2, http://tpia.teaplants.cn/). Ultimately, 34 candidate genes with higher expression levels in the 340 kb region covered by the above 16 QTLs were pinpointed (Table S3, see online supplementary material).

### Identification of droopy leaves related gene, *CsEXL3,* based on conjoint analysis of GWAS and RNA-seq

**Figure 2:**
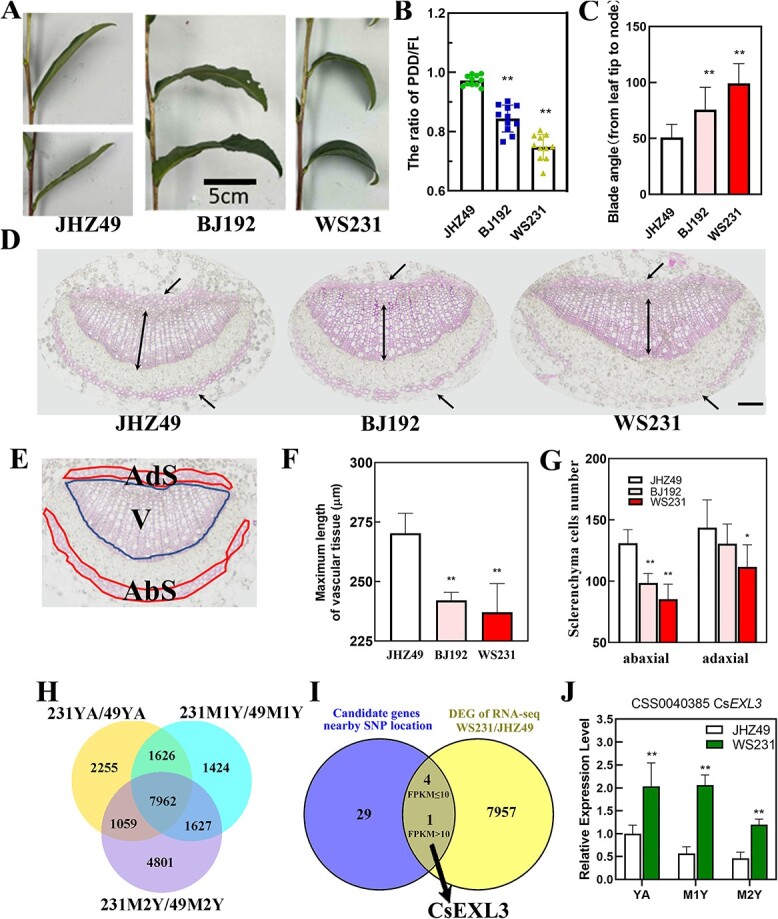
Identification of droopy leaves related gene, *CsEXL3*, in tea plant germplasm. (**A**) Representative leaves picture of tea plant germplasm with straight or droopy blade. Scale bar represents 5 cm. (**B**) The PF of selected germplasm with straight or droopy blade. Data shown represent the means ±SD of at least ten biological replicates. (**C**) The blade angle of selected germplasm with straight or droopy blade. (**D**) Lignin staining on cross-sections of the middle part in leaf blades midrib with phloroglucinol-HCl. The middle parts were selected on the direction from tip to the blade-stem boundary. The upper or lower unidirectional arrow indicates adaxial or abaxial sclerenchyma cells, respectively. Double arrow indicates the maximum length of vascular tissue. Scale bar represents 100 μm. (**E**) Diagram illustrating the adaxial sclerenchyma cells (Ads), abaxial sclerenchyma cells (Abs), and vascular bundle (V) in the leaf blades midrib of tea plant. (**F**) The maximum length of vascular tissue in the leaf blades midrib of selected germplasm. (**G**) The number of adaxial and abaxial sclerenchyma cells in the leaf blades midrib of selected germplasm. (**H**) Venn diagram showing the number of common differentially expressing genes (DEGs) in three type tissue of compared germplasms, JHZ49 and WS231. YA, the apical bud and first leaf; M1Y, first mature leaves; M2Y, second mature leaves. (**I**) Venn diagram showing the number of common genes between candidate genes from GWAS and DEGs of RNA-seq in WS231 and JHZ49. The common genes were divided into two groups, genes with FPKM≥10 and genes with FPKM<10. (**J**) The relative expression level of *CsEXL3* in three type tissue of WS231 and JHZ49. **P < 0.01; *P < 0.05.

To refine the selection of potential candidate genes, transcriptomes of JHZ49 (with a higher PF) and WS231 (with a lower PF) were used for further comparison (Fig. 2A and B). The blade angle and midrib section of accession with higher PF (JHZ49) as well as accession with lower PF (BJ192 and WS231) were measured and observed, respectively (Fig. 2C to G). JHZ49 had straight leaves with a PF value of almost one and a smaller blade angle of 50.99°. BJ192 had droopy leaves with a lower PF value and a larger blade angle of 75.64° while compared to JHZ49. WS231 had droopy leaves with the lowest PF value and larger blade angle of 99.16° while compared to JHZ49 or BJ192 (Fig. 2B and C).

Leaf droopiness and larger blade angle were attributed to abnormal cell division in the midrib as previously reported [5]. Lignin staining on cross-sections showed that lignin mainly accumulated in the adaxial sclerenchyma cells (Ads), abaxial sclerenchyma cells (Abs), and vascular bundle (V) of the tea plant’s leaf blades midrib (Fig. 2D and E). Relative to JHZ49, both BJ192 and WS231 demonstrated a reduced maximum length of vascular tissue (Fig. 2F) and a decreased cell number in the abaxial sclerenchyma (Fig. 2G). Additionally, the vascular tissue area of these accessions has been determined (Fig. S1, see online supplementary material). Moreover, WS231 but not BJ192 had fewer adaxial sclerenchyma cell numbers (Fig. 2G). Considering that WS231 had the lowest PF value as well as a significant difference in both vascular tissue length, abaxial and adaxial sclerenchyma cell numbers, the apical bud and first leaf (YA); first mature leaves (M1Y), and second mature leaves (M2Y) of WS231 were chosen for further transcriptional analysis against its control accession, JHZ49 (Fig. S2, see online supplementary material).

Significant differentially expressed genes (DEGs, *P* < 0.05) were extracted from RNA-seq in YA, M1Y, and M2Y of WS231 and JHZ49 (Fig. 2H; Table S4, see online supplementary material). A total of 7962 DEGs were identified in all three types of leaf tissue as the overlapped area shown (Fig. 2H). Results of GO (Figs S3 and S4, see online supplementary material) and KEGG (Fig. S5 and Table S5, see online supplementary material) analyses indicated that there are no enrichments in lignin metabolic or cell proliferation-related GO or KEGG pathways in tested three tissues, YA, M1Y, and M2Y. However, one cell proliferation-related gene, *PSK* (*PHYTOSULFOKINE*, novel.9663), was highly expressed in all three tissues of JHZ49 but absent in WS231 (Table S4, see online supplementary material). From the pool between these 7962 DEGs and 34 candidate genes from GWAS, 5 overlapped genes were chosen for further analysis, including *CsEXL3*, *FYVE1* (*FAB1, YOTB, VAC1, AND EEA1 DOMAIN-CONTAINING 1*), *UGD1* (*UDP-GLUCURONIC ACID DECARBOXYLASE 1*), *DL* (*DNA LIGASE*), and *PL4* (*PECTATE LYASE 4-LIKE*) (Fig. 2I; Table S6, see online supplementary material). Among these genes, only the FPKM value of *CsEXL3*, which indicates the expressing level, was above 10 in all sample sets, while that of the other four genes fell below 10 (Fig. 2I). The expression levels of *FYVE1*, *UGD1*, and *DL* in all three types of tissue from WS231 were significantly lower than that of JHZ49. The expression levels of *PL4* in YA and M1Y of WS231 were significantly lower than those of JHZ49 but the expression level of *PL4* was almost the same in M2Y of WS231 as that of M2Y from JHZ49. *CsEXL3* was located within 0.71 Mb of three lead SNPs (q1.2SNP1, q1.2SNP2, and q1.2SNP3) corresponding to QTL site, q1.2, on chromosome 1 (Table S2; Fig. S6, see online supplementary material). Several SNP sites inside *CsEXL3* exon had high correlation with both three lead SNPs. *CsEXL3* belongs to the EXO/EXL family and has the closest genetic relationship with EXO like 3 but not EXO (EXORDIUM), a gene regulated by BR and associated with development [11] (Fig. S7, see online supplementary material).

Previous reports have identified *EXP5* and *KCS1* as EXO-responsive genes [11]. Our analysis of these genes’ homologs in the tea plant, including *CsEXP5.1*, *CsEXP5.2*, and *CsKCS1* (Fig. S8, see online supplementary material), revealed that the relative expression levels of *CsEXP5.1* and *CsEXP5.2* were down-regulated in YA, M1Y, and M2Y of WS231 when compared to JHZ49. The relative expression levels of *CsKCS1* were down-regulated only in M2Y of WS231 when compared to JHZ49. The expression pattern of these genes was contrary to higher expressing levels of *EXP5* and *KCS1* in the overexpressing line of *AtEXO* [10], indicating that CsEXL3 had different functions with the conserved regulating function of EXO.

The expression level of *CsEXL3* was further measured via qRT-PCR in YA, M1Y, and M2Y of ‘Wushi’(WS231) and ‘Jianghongzhong’ (JHZ49). The expression level of *CsEXL3* was higher in WS231 with droopy leaves than in JHZ49 with straight leaves (Fig. 2J), which suggests that CsEXL3 might positively regulate droopiness in tea leaves. Therefore, the function of *CsEXL3* was further determined in the tea plant to verify the hypothesis that CsEXL3 positively regulated droopiness.

### *CsEXL3* promoted leaf droopiness of tea plant in transcriptional level

**Figure 3:**
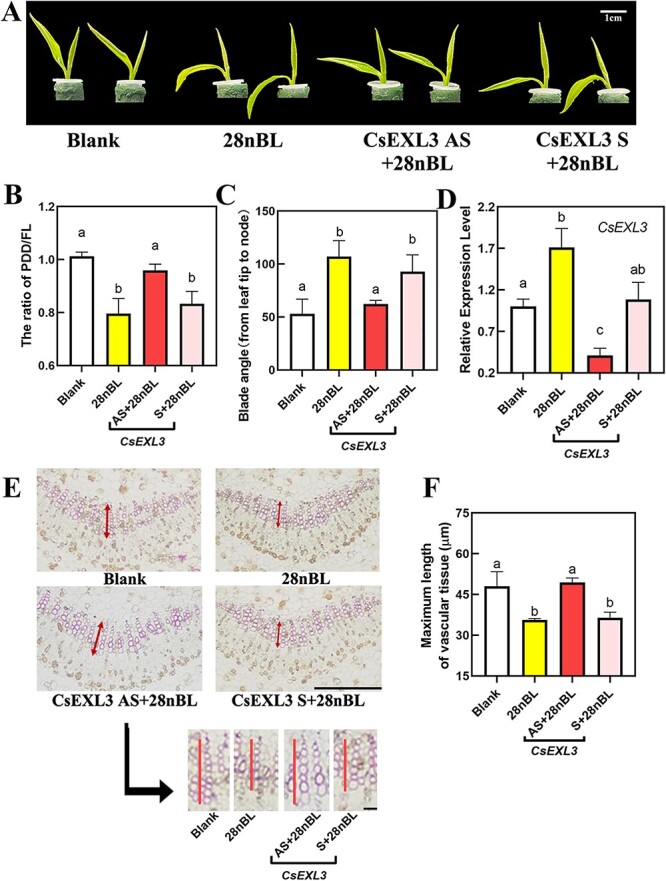
Morphological characterization, physiological characterization and related gene expression levels of *CsEXL3*-silencing tea plant. (**A**) Representative pictures of *CsEXL3*-silencing tea plant. Blank, blank control. 28nBL, control group. CsEXL3 AS, experimental group for silencing *CsEXL3* expression. CsEXL3 S, negative control. Scale bar represents 1 cm. (**B**) The ratio of PDD/FL of *CsEXL3*-silencing tea plant. (**C**) Blade angle of *CsEXL3*-silencing tea plant. (**D**) Relative expression level of CsEXL3 in *CsEXL3*-silencing tea plant. (**E**) Lignin staining on cross-sections of the middle part in leaf blades midrib of *CsEXL3*-silencing tea plant with phloroglucinol-HCl. Scale bar represents 100 μm. Pictures of vascular tissue with maximum length were shown as blank arrow indicated. Red vertical line indicated the maximum length. Shorter scale bar represents 10 μm. (**F**) Maximum length of vascular tissue in *CsEXL3*-silencing tea plant. For (**B**) and (**C**), values are means ± SD of six biological replicates. For (**D**) and (**F**), values are means ± SD of four biological replicates. Statistical analysis was performed with ANOVA. Bars with different letters indicate significant difference (*P* < 0.05).

To figure out the regulating relationship of CsEXL3 in droopiness, the expression of *CsEXL3* in tea plant bud was silenced by using antisense oligonucleotides (AsODN). For conventional AsODN processing methods, the leaves of tea plant bud could not bend spontaneously without additional treatments in a short time of 5 days. Given that *EXO* exhibits responsiveness to exogenous BR treatment, we inferred that exogenous BR treatment might induce the leaf droopiness of tea plant for a brief period during the AsODN experiment of *CsEXL3*. In this study, 28nBL was found to induce droopiness of the bud leaves in tea plant during the short-term AsODN experiment (Fig. 3A). 28nBL treatment significantly induced leaf droopiness in tea plants with decreased PF and uplifted blade angle (Fig. 3B and C). However, the droopiness caused by exogenous BR treatment was hampered in *CsEXL3*-silencing with AsODN (CsEXL3 AS) group. The PF of CsEXL3 AS was significantly increased and the blade angle of CsEXL3 AS was significantly reduced when compared to control (28nBL) and negative control (CsEXL3 S) (Fig. 3B and C). Subsequent analysis of the relative expression level of *CsEXL3* confirmed the effect of AsODN and established a correlation between droopiness and the expression pattern of *CsEXL3*. The expression level of *CsEXL3* was increased by 1.5-fold with 28nBL treatment (Fig. 3D) and this 28nBL-induced expression level of *CsEXL3* was significantly knocked down with AsODN of CsEXL3 in the CsEXL3 AS group. The expression level of CsEXL3 in the CsEXL3 AS group was even lower than the blank group, which confirmed the efficient suppressing effect of AsODN on the expression level of *CsEXL3* (Fig. 3D). The inhibited leaf droopiness in the *CsEXL3*-silencing tea plant was consistent with the promoted blade bending degree in WS231 with the higher expressing level of *CsEXL3* (Fig. 2J).

Lignin staining on leaf blades midrib cross-sections of *CsEXL3*-silencing tea plant was conducted to explore droopiness-related variation at the cellular level. The maximum length of vascular tissue was decreased in 28nBL-treated tea plant. Meanwhile, the maximum length of vascular tissue in the *CsEXL3*-silencing tea plant was significantly longer than the control or negative control (Fig. 3E and F), which was in accordance with higher *CsEXL3* expressing level and shorter maximum vascular tissue length in droopy leaves of WS231 as well as lower *CsEXL3* expressing level and longer maximum vascular tissue length in the JHZ49. In total, high transcriptional levels of *CsEXL3* result in shorter maximum vascular tissue length and then promote leaf droopiness of tea plant. However, the molecular mechanism of transcriptional activation on *CsEXL3* needs to be revealed.

### CsBES1.2 promoted leaf droopiness of tea plant through up-regulating the expression of *CsEXL3*

As the transcriptional expression of *CsEXL3* was responsible for regulating droopy leaves, it is crucial to identify the upstream regulator of transcriptional activation on *CsEXL3* to reveal the molecular mechanism. BES1 has been proven to transcriptionally regulate the expression of target genes to reflect the roles of BR in growth and development [17, 18]. Hence, the transcriptional activation of CsBES1.2 on *CsEXL3*, as induced by exogenous BR treatment leading to leaf droopiness, was investigated.

**Figure 4:**
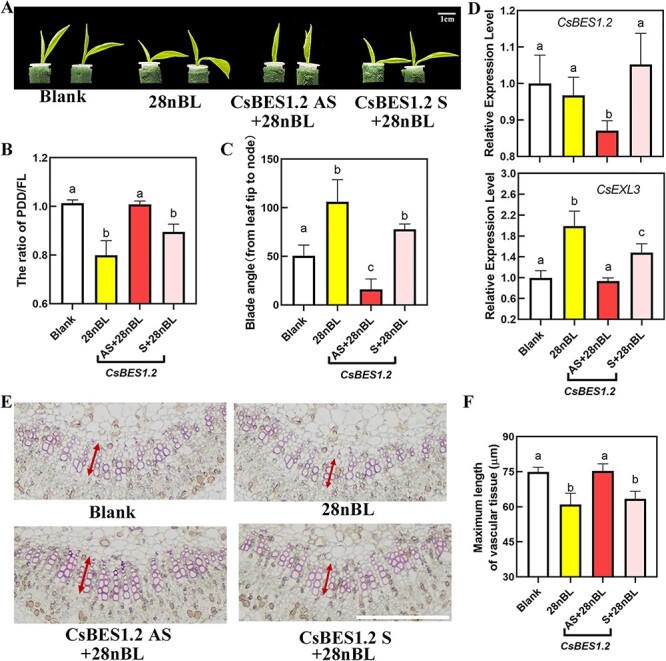
Morphological characterization, physiological characterization, and related gene expression levels of *CsBES1.2*-silencing tea plant. (**A**) Representative pictures of *CsBES1.2*-silencing tea plant. Blank, blank control. 28nBL, control group. CsBES1.2 AS, experimental group for silencing *CsBES1.2* expression. CsBES1.2 S, negative control. Scale bar represents 1 cm. (**B**) The ratio of PDD/FL of *CsBES1.2*-silencing tea plant. (**C**) Blade angle of *CsBES1.2*-silencing tea plant. (**D**) Relative expression level of CsBES1.2 in *CsBES1.2*-silencing tea plant. (**E**) Lignin staining on cross-sections of the middle part in leaf blades midrib of *CsBES1.2*-silencing tea plant with phloroglucinol-HCl. The white line indicates a scale bar that represents 100 μm. (**F**) Maximum length of vascular tissue in *CsBES1.2*-silencing tea plant. For (**B**) and (**C**), values are means ± SD of six biological replicates. For (**D**) and (**F**), values are means ± SD of four biological replicates. Statistical analysis was performed with ANOVA. Bars with different letters indicate significant difference (*P* < 0.05).

If CsBES1.2 serves as the upstream transcriptional factor of *CsEXL3*, CsBES1.2 might participate in regulating leaf droopiness. In this study, initial confirmation of CsBES1.2’s function in leaf droopiness was achieved by silencing *CsBES1.2*, which significantly counteracted 28nBL-induced leaf droopiness (Fig. 4). The inhibition of silencing *CsBES1.2* in droopiness was even stronger than that of *CsEXL3* in tea plants. The PF of CsBES1.2 AS was significantly uplifted and the blade angle of CsBES1.2 AS was reduced at the same time when compared to the control group or negative control. More importantly, the expression level of both *CsBES1.2* and *CsEXL3* were down-regulated in the *CsBES1.2*-silencing tea plant, indicating that CsBES1.2 might positively regulate the expression of *CsEXL3*. RNA-seq results also indicated that the uplifted expressing level of *CsBES1.2* in WS231 than JHZ49, corresponding to the higher expressing level of *CsEXL3* in WS231 (Fig. S9, see online supplementary material).

Then, the lignin stain suggested that the maximum length of vascular in the *CsBES1.2*-silencing tea plant was longer than the control (Fig. 4), which was the same as that of the *CsEXL3*-silencing tea plant or the JHZ49 with lower expressing levels of both *CsBES1.2* and *CsEXL3*. These results indicated that CsBES1.2 promoted leaf droopiness of tea plants by up-regulating the expression of *CsEXL3* and inhibited the maximum length of vascular tissue.

### CsBES1.2 transcription regulated the expression of *CsEXL3* via binding E-box in the promoter of *CsEXL3*

**Figure 5:**
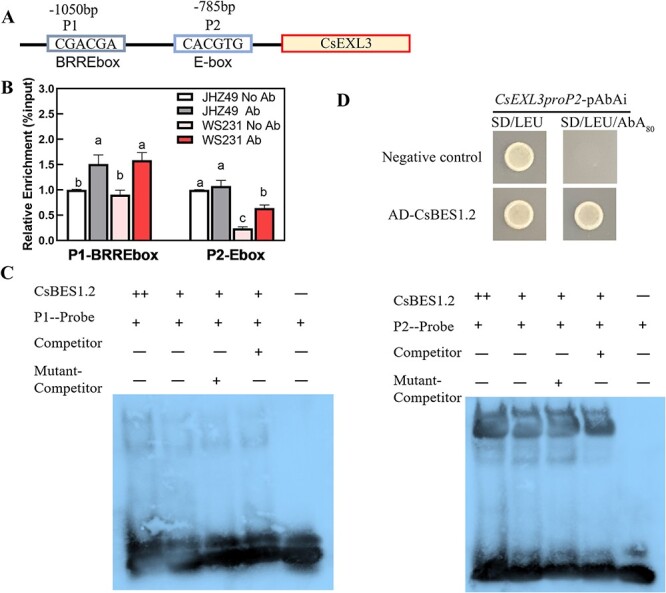
CsBES1.2 transcriptional regulated the expression of *CsEXL3*. (**A**) Schematic diagram of CsEXL3 and its promoters, indicating the amplicons used for ChIP-qPCR. Positions of BRRE-box (BR response element, P1) and E-box (P2) are indicated. (**B**) ChIP-qPCR assays showing that CsBES1.2 was associated with the locus of CsEXL3. Chromatin of tea plants expressing CsBES1.2 was immunoprecipitated with anti-CsBES1.2 polyclonal antibodies, and the result of *CsGAPDH* served as a control. The relative enrichment for ChIP signal was displayed as the percentage of total input DNA. Values are means ± SD of three biological replicates. Statistical analysis was performed with ANOVA. Bars with different letters indicate significant difference (*P* < 0.05). (**C**) DNA electrophoretic mobility shift assay (EMSA) showing that the binding of CsBES1.2-MBP to the BRRE-box (P1) and E-box (P2) of *CsEXL3* promoter in vitro. Biotin-labeled probes were incubated with CsBES1.2-MBP and the free and bound DNAs were separated on an acrylamide gel. (**D**) Yeast one-hybrid analysis showing that CsBES1.2 binds to the promoter of *CsEXL3*. 80 ng/mL Aureobasidin A (AbA), a yeast cell growth inhibitor, was used as a screening marker. The empty vector and the *CsEXL3* promoter containing E-box (P2) were used as negative controls.

To uncover the details of the transcriptional regulation of CsBES1.2 on *CsEXL3*, we carried out further independent experiments to prove the transcriptional activation of CsBES1.2. We first cloned CsBES1.2, one of the BZR/BES1 family members according to the phylogenetic tree (Fig. S10, see online supplementary material), from JHZ49 or WS231 to confirm the authentic existence of CsBES1.2. Two binding sites, BRRE-box (P1) and E-box (P2), were located at -1050 bp and -785 bp of *CsEXL3* promoter by the JASPAR database, respectively (http://jaspar.genereg.net/) (Fig. 5A).

ChIP-seq of CsBES1.2 in WS231 confirmed that the binding region of CsBES1.2 is located on the promoter of *CsEXL3* in tea plants (Fig. S11, see online supplementary material). A total of 12 906 peaks for identified genes were summarized and 27.92% of these peaks were distributed inside the 3 kb upstream of genes (Fig. S11A and B, see online supplementary material). Specifically, the peak for the binding site of CsBES1.2 on the promoter region of *CsEXL3* was also identified in the ChIP-seq of CsBES1.2 (Fig. S11C, see online supplementary material). ChIP-qPCR results suggested that enrichment of CsBES1.2 bound to these two types of box (Fig. 5B). The enrichment of CsBES1.2 binding to BRRE-box was significant in both JHZ49 and WS231. However, the enrichment of CsBES1.2 binding to E-box was more significant in WS231 than in JHZ49, indicating the binding of E-box was more specific than that of BRRE-box in WS231.

EMSA was then conducted with purified CsBES1.2-MBP and a 20-bp DNA probe containing BRRE-box or E-box (Fig. 5C). CsBES1.2-MBP bound to the BRRE-box and E-box. The binding of CsBES1.2-MBP on the E-box could be outcompeted by the unlabeled DNA probe but not the probe without the E-box. This competition on the P1 probe containing BRRE-box between the competitor and CsBES1.2-MBP was not very clear. The binding of CsBES1.2-MBP on the E-box was stronger than that of the BRRE-box because the band intensity of the bound P1 probe was darker than that bound P2 probe.

Furthermore, the yeast one-hybrid (Y1H) assay was performed to test the interaction between CsBES1.2 and *CsEXL3* promoter (Fig. 5D). Yeast cells co-transformed with pGADT7-CsBES1.2 and *CsEXL3proP2*pAbAi containing E-box grew on synthetic defined (SD)/−Leu medium containing 80 ng/mL aureobasidin A (AbA), whereas negative control cells co-transformed with the empty pGADT7 vector and *CsEXL3proP2*pAbAi did not, confirming the interaction of CsBES1.2 and the *CsEXL3* promoter in yeast. Y1H assay for CsBES1.2 and the P1 location of *CsEXL3* promoter failed for the self-activation of *CsEXL3proP1*pAbAi containing BRRE-box could grow on SD/−Leu containing 0 ng/mL to 1500 ng/mL AbA. These interaction-related experiments indicated the binding of CsBES1.2 on the E-box but not the BRRE-box.

### CsEXL3 down-regulated lignin content and the expression levels of lignin biosynthesis-related genes

**Figure 6:**
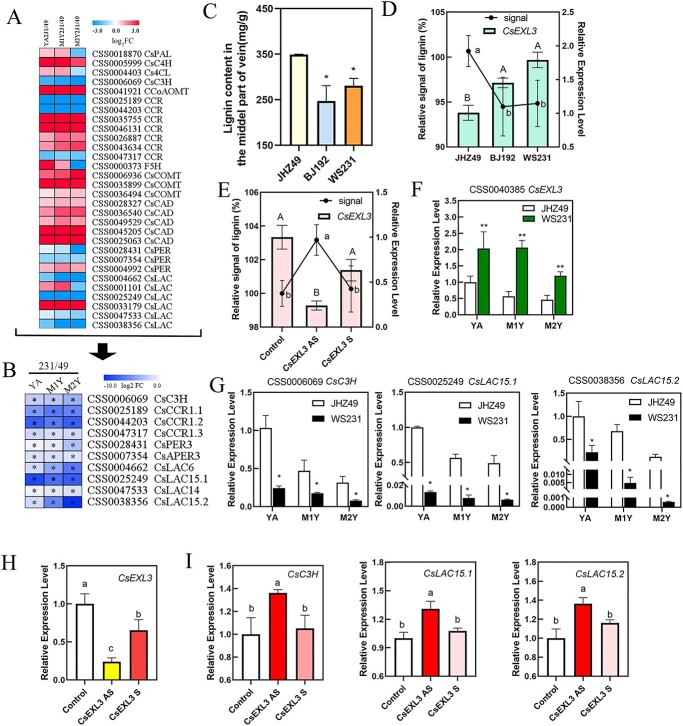
CsEXL3 regulated lignin content and the expression levels of lignin biosynthesis genes. (**A**) The expression level heatmap of lignin biosynthesis genes from RNA-seq in JHZ49 and WS231. (**B**) The heatmap of lignin biosynthesis genes that significantly differentially expressing in different tissue of JHZ49 and WS231. (*P < 0.05). (**C**) The lignin content in the middle part of three tea plant cultivars’ veins. (**D**) Relative lignin signal and the relative expression level of *CsEXL3* in leaves of three tea plant cultivars, JHZ49, BJ192, and WS231. (**E**) Relative lignin signal and the relative expression level of *CsEXL3* in leaves of *CsEXL3*-silencing tea plant. (**F**) The relative expression level of *CsEXL3* in different tissue of JHZ49 and WS231. (**G**) The relative expression level of lignin biosynthesis genes, *CsC3H*, *CsLAC15.1*, and *CsLAC15.2*, in different tissues of JHZ49 and WS231. (**H**) The relative expression level of *CsEXL3* in *CsEXL3*-silencing tea plant. (**I**) The relative expression level of lignin biosynthesis genes, *CsC3H*, *CsLAC15.1*, and *CsLAC15.2*, in *CsEXL3*-silencing tea plant. Values are means ± SD of three biological replicates. Statistical analysis was performed with ANOVA. Bars with asterisks or different letters indicate significant difference (*P* < 0.05).

CsEXL3 participated in regulating the leaf droopiness not only through regulating vascular bundle length in the midrib of tea plant but also might regulate the lignin content of leaves by affecting lignin biosynthesis genes. We summarized the expression pattern of all known lignin biosynthesis genes from RNA-seq in WS231 and JHZ49 (Fig. 6A). Significant differentially expressed lignin biosynthesis genes were then listed (Fig. 6B). Lignin metabolic-related DEGs, including *CsC3H*, *CsCCRs*, and *CsLACs*, were all down-regulated in droopy leaves of WS231 when compared to straight leaves of JHZ49 (Fig. 6B). Meanwhile, the contents of lignin in mature leaves were decreased in the middle part of the vein (the same location of the tissue staining section as Fig. 2D shown) in BJ192 and WS231 than in JHZ49 (Fig. 6C). However, the lignin content in the whole droopy leaves of BJ192 and WS231 was more insignificantly decreased than that in the whole straight leaves of JHZ49 (Fig. S12, see online supplementary material). These results indicate that the primary difference in lignin accumulation between droopy and straight leaves occurs in the middle part of the vein. For the determination of lignin leaves in younger leaves, the relative signal for metabolite stain was an effective and accurate way to compare metabolite content in different samples [18] because sclerenchyma cells and vascular bundle were not fully developed and the lignin contents of these cells were at a relatively lower level for the spectrophotometer. The relative lignin signals in BJ192 and WS231 were weaker than JHZ49 and the expressing levels of *CsEXL3* in BJ192 and WS231 were stronger than JHZ49 (Fig. 6D), which indicates a negative correlation between the lignin accumulation and the expressing level of *CsEXL3*. To confirm this negative correlation, the relative lignin signal in *CsEXL3*-silencing tea plant was also detected (Fig. 6E). The relative lignin signal was more altered in *CsEXL3*-silencing tea plant than that of the control (Fig. 6E), which was in accordance with the negative correlation between the lignin accumulation and the expressing level of *CsEXL3*. However, the relative lignin signal in *CsBES1.2*-silencing tea plant was at the same level of control (Fig. S13, see online supplementary material), suggesting that CsBES1.2 did not participate in CsEXL3-regulated lignin accumulation in the midrib.

**Figure 7:**
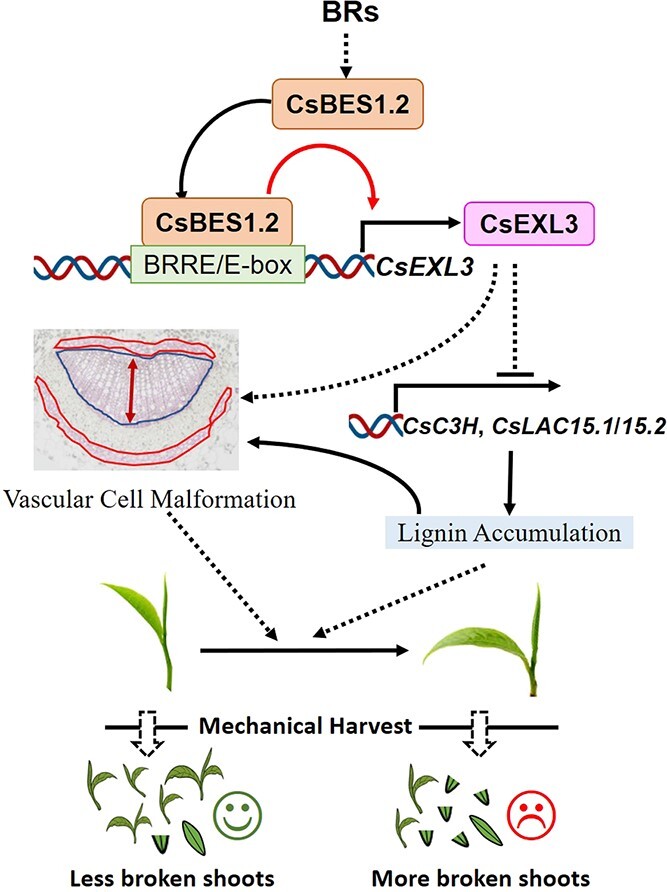
Schematic representation of relationship between transcriptional regulation of CsBES1.2 on *CsEXL3* and leaf droopiness. The effects of CsEXL3 on the vascular cell malformation, the expression levels of lignin biosynthesis genes and lignin content are also shown.

Meanwhile, only three lignin biosynthesis genes (*CsC3H*, *CsLAC15.1*, and *CsLAC15.2*) have the lower expressing levels in YA, M1Y, and M2Y of WS231 according to qRT-PCR, which corresponded to the higher expressing levels of *CsEXL3* (Fig. 6F and G). This phenomenon suggested that CsEXL3 reduced the expressing levels of *CsC3H* and *CsLACs*. The same negative regulation was also observed in *CsEXL3*-silencing tea plant in the opposite way. The expressing levels of *CsC3H* and *CsLACs* were significantly increased in *CsEXL3*-silencing tea plant (Fig. 6H and I). To sum up, CsEXL3 negatively regulated lignin content in the midrib of tea plant leaves and down-regulated the expressing levels of lignin biosynthesis genes, *CsC3H* and *CsLACs* (Fig. 7).

## Discussion

### The similar and different roles of EXO/EXL family genes from different clades of the phylogenetic tree

*EXLs*, integral members of the EXO/EXL gene family, share a similarity range of 37.54 to 78.76% similarity with EXO in *Arabidopsis* [19] (Fig. S2, see online supplementary material). This resemblance primarily stems from the PHOSPHATE INDUCED-1 (PHI-1) motif, which was first discovered in the cultured phosphate-starved cells of tobacco [20]. However, the biomass of young leaves in tea plant was not changed in a phosphate-deficiency environment [21] and none of the leaf architecture-related symptoms were found under phosphate-starved conditions [22], which indicates motif PHI-1 in tea plants had no relationship with phosphate starvation. Furthermore, no significant expression of EXO/EXL family genes was observed in tea plants under phosphate treatment [21], indicating their non-responsiveness to phosphate conditions.

EXOs/EXLs family encompasses nine conserved motifs (I-IX), including motif PHI-1. The homologous protein of AtEXL3 in rice (Os06g04250) [9] lacks motif I when compared to EXO proteins. The motif I in this gene, which contained 29 amino acid bases, was replaced with another unidentified motif, which indicates a distinct function for EXL. This different function might be related to tissue localization according to proteome in *Arabidopsis* and *Physcomitrium patens*. PsEXO1 was localized in plasmodesmata of *P. patens* and divided into Clade III of the phylogenetic tree and Clade III also contained AtEXO, AtEXL1, and AtEXL2 from *Arabidopsis* [23]. AtEXL3, AtEXL5, and AtEXL7 were divided into Clade II [23] and *CsEXL3* was a homologous gene of *AtEXL3* (Fig. S7, see online supplementary material). AtEXL3 was only identified from proteomes of *Arabidopsis* cell wall instead of plasmodesmata [24]. Homologous proteins of AtEXL3 were also not figured out in the plasmodesmata proteomes of *P. patens*. This evidence indicated the conserved localization of EXL3 family proteins in cell walls but not plasmodesmata.

Differences in motifs across various Clades of EXL/EXO also suggested a functional divergence among EXO family genes in leaf development and growth [12, 19]. For instance, GhEXO2, the homologous gene of AtEXL2, and GhEXO7 belong to Clade III and positively regulated leaf size in cotton [12, 25] whereas CsEXL3, the homologous gene of AtEXL3 belonging to Clade II, regulated leaf droopiness instead of leaf size. Meanwhile, EXO/EXL from the same Clade had similar and different functions. For example, AtEXL1 and AtEXO were both identified as BR-induced genes from Clade III but executed different functions. AtEXL1 controlled growth and development under carbon starvation conditions but its related mutants did not show any ability under the energy-sufficient environment [19]. CsEXL3 positively regulated leaf droopiness but droopiness-related traits had not been reported in AtEXL3 mutant (*AtEXL3*, At5g51550 information on TAIR website, www.arabidopsis.org), which was different from the conserved function of EXL1 and EXL2. All in all, the function of CsEXL3 in regulating leaf droopiness broadens cognition about diverse roles of EXO/EXL family genes across different Clades of the phylogenetic tree.

### The role of BR-related gene in breeding improvement of tea plant

Former studies and our results concerned the abilities of BR on various impacts of breeding improvement in the tea plant, such as health-promoting metabolites accumulation, biotic stress resistance, growth and development [26–29] (Figs 3and 4). Components from the BR signaling pathway and BR responsive genes were both identified to fulfill a vital role in these impacts. For BR signaling pathway components, BRI1-like 2 was the homologous gene of the BR receptor and might participate in the vegetative growth of tea plants according to the genomic comparison between *C. sinensis var. assamica* with larger leaves and *C. sinensis var. sinensis* with smaller leaves according to the 200 K SNP array [27]. The expression levels of *BAK1* (BR co-receptor) and *BIM1* (the interaction factor of *BES1*) were uplifted after exogenous BR treatment and therefore enlarged thylakoids size in leaf chloroplasts of tea plants [29]. Our results also suggested that the key BR signaling pathway component, CsBES1.2, was involved in regulating the leaf droopiness of the tea plant (Fig. 4). For BR-responsive genes in tea plants, *CsMYB4-like* partook in lignin accumulation in response to disease through possible transcriptional activation on the expression of lignin metabolic genes [28]. Our result indicated that CsEXL3 acted as a BR-responsive gene (Fig. 3) and affected lignin accumulation as well as the expression levels of lignin biosynthesis genes in tea plants (Fig. 6), thereby enriching the intricate relationship between phytohormones and agricultural production measures in tea industry [30]. The droopiness trait of the CsEXL3-silence tea plant also proved that CsEXL3 participates in the diverse genes-involved regulation networks on leaf development of tea plant [31] (Fig. 3). Altogether, BR signaling pathway components, such as BES1 family genes, and BR responsive genes, such as EXO/EXL family genes, both have huge potential to accelerate the progress of breeding improvement in tea plants.

## Materials and methods

### Plant materials and growth conditions

We evaluated 331 tea accessions (*C. sinensis* (L.) O. Kuntze) in this study from National Tea Germplasm Repository Hangzhou (NTGRH) at the Tea Research Institute of the Chinese Academy of Agricultural Sciences located at Hangzhou, Zhejiang, China. After photography and measurement of PDD/FL ratio, leaves of selected accessions were frozen in liquid nitrogen as samples and then stored at −80°C for further testing.

### Measurement of PDD/FL ratio

The attached leaves of 331 accessions in NTGRH were selected to be photographed for the determination of curling degree. The curling degree of the leaf blade was measured via the PDD (proximal-distal distance) / FL (full length of a leaf blade) ratio. After photographing tea plant shoots, the pictures were measured by ImagJ to get the length of PDD or FL and then to calculate the ratio of PDD/FL.

### Anatomical observation of tea plant leaf

Droopy blades of BJ192 and WS231 or straight blades of JHZ49 (second or third stalk position from the top) were selected (Fig. 2) and the cross-section in the middle of leaf midrib was cut with a scalpel. Leaf blades from the apical bud and the first leaf of JHZ49 used for chemical treatment and gene suppression were collected (Figs 3 and 4) and cut in the same section with a scalpel. Then, the selected sections were fixed in FAA solution at 4°C overnight, dehydrated in graded ethanol from 10% to 100% and embedded. Polylysine coated slides were used to apply 10 μm sections. The sections were stained with toluidine blue or 1% phloroglucinol (w/v) in 12% HCl. For toluidine blue staining, the sections were heat fixed and stained for photography by a bright-field microscope, ECLIPSE Ci (Nikon, Tokyo, Japan). For phloroglucinol-HCl staining, the sections were stained for 5 min and immediately observed with a light microscope (Nikon, Tokyo, Japan). For the signal of lignin, the shade of phloroglucinol-HCl stained lignin could represent the relative contents of lignin. The shade signals of phloroglucinol-HCl stained lignin images were calculated through the software ImageJ.

### Genome-wide association study

The tea plant TeaGVD database was used to obtain the SNPs inside the genome sequence of 146 accessions from 331 evaluated germplasms in NTGRH [32]. The detailed information of 146 accessions is listed in Table S7 (see online supplementary material). ‘Shachazao’ 2 was used as the reference genomic data. The PDD / FL ratio of these tea plants was set as the index for the degree of blade bending. The resulting SNPs were then used to perform a genome-wide association study analysis for the PDD/FL ratio under a mixed linear model implemented in the EMMAX program. *P*-value thresholds were calculated after Bonferroni correction [33]. The threshold of significant candidate loci (Lead SNPs) was determined by GEC software. Genes inside the fragment of significant SNP ± 0.5 Mbp were collected and their transcriptional levels in different tissues of tea plant, especially in leaf, were predicted in the ‘Shuchazao’ V2 database from TeaGVD. Genes with a high transcriptional level (threshold >20) were selected as candidate genes.

### Cloning and vector constructs

Cloning and plasmid constructs were generated following standard molecular biology protocols. cDNAs from the apical bud and first leaf of ‘Shuchazao’ were used as templates for cloning. The full-length coding sequences of CsEXL3 and CsBES1.2 with or without termination codon were amplified via PCR with KOD plus Neo and inserted into pEASY-Blunt Zero Cloning Vector. The cloned coding sequences of *CsEXL3* and *CsBES1.2* were confirmed by sequencing. The recombinant vector, pEASY-CsBES1.2, was used as the template to amplify the full-length region for inserting into pMAL-c5x. Used primers in this experiment are listed in Table S8 (see online supplementary material).

### RNA extraction and quantitative real-time PCR

For RNA extraction, 0.2 g of selected leaves were frozen in liquid nitrogen and then ground into a powder with a liquid nitrogen-cooled can in TissueLyser II. The powder was mixed with 1 mL RNAiso plus according to the manufacturer’s instruction (Takara, Kusatsu, Japan), and then RNA was reverse-transcribed into cDNA using PrimeScript RT reagent with gDNA Eraser (Takara, Kusatsu, Japan). cDNAs were further used as templates for quantitative real-time PCR (qRT-PCR).

For qRT-PCR, SYBR Green (Roche, Basel, Switzerland) was then applied in Lightcycler 480 II (Roche, Basel, Switzerland). Primers for candidate genes in this experiment are listed in Table S8 (see online supplementary material).

### RNA-seq

The tea cultivars, JHZ49 and WS231, were collected from their original regions and cultivated at the NTGRH since 2006. Tender tissue (the apical bud and first leaf, YA), first mature leaves (from top to bottom, M1Y) and second mature leaves (M2Y) of JHZ49 or WS231 were collected to extract total RNA. The sketch map for the position of YA, M1Y, and M2Y on the tea plant is shown in Fig. S2 (see online supplementary material). Illumina MiSeq library was constructed as described by manufacturer’s instructions (Illumina, San Diego, CA, USA) and then sequenced with the Illumina NovaSeq platform.

### Determination of lignin content

The extraction and measurement of total lignin in tea leaves were performed as previously described [28] with modification. Tea leaves (mature leaves on second or third stalk position from the top) were dried at 80°C for 5 days. Leaf veins were separated from mesophyll tissue as different samples. After grounding into powder with 5.5 mm steel ball in grinding jar, 0.02 g sample was mixed with 1750 μl distilled water and then centrifuged at 10000 rpm for 5 min. Supernatant was dissolved with 1750 μl methanol and the mixture was boiled at 60°C for 20 min. The mixture was centrifuged at 10000 rpm for 5 min to get the precipitate after cooling to room temperature in ice. The precipitate was blow dried by gaseous nitrogen. The dried precipitate was dissolved with 1 mL 3 M HCl and 100 μL thioglycolic acid and then heated at 80°C for 3 h, followed by cooling in ice. After centrifuge at 10000 rpm for 5 min, sediment was washed with water and dissolved with 1 M NaOH at 80 rpm for 16 h. Then, the mixture was centrifuged at 10000 rpm for 5 min to collect supernatant. Collected supernatant was added with 200 μL 12 M HCl and then cooled in ice for 4 h. After centrifuge at 10000 rpm for 5 min, the precipitate was dissolved with 1 M NaOH as the sample for lignin measurement. Then, 200 μL sample was diluted with 1800 μL 1 M NaOH and the content of total lignin detected at OD280 by spectrophotometer with 1 M NaOH set as blank control and alkali lignin (Sigma-Aldrich) used for establishment of the standard curve. Three biological replicates were analysed for each sample.

### Chemical treatment and gene suppression in tea plants

The 28-nBL (28-homobrassinolide) was used to induce blade curving in the direction of leaf vein. For exogenous BR treatment, tender tissue (the apical bud and first leaf) of JHZ49 were harvested and grown in 5 μM 28-nBL solution under a 16 h photoperiod (22/28°C, night/day). Water without any solution was treated as blank control. In order to suppress target gene expression, CsEXL3 or CsBES1.2 antisense oligonucleotides (AsODN) were designed as previous research described [34] and 20 μM AsODN of CsEXL3 or CsBES1.2 were added in 5 μM 28-nBL solution for treatment of tender tissue. Random nonsense ODNs were treated as the control. After 5 days treatment, the samples of treated tender tissue were photographed, selected for anatomical observation, frozen in liquid nitrogen and stored in −80°C. Six biological replicates were analysed for each sample.

### ChIP-qPCR and ChIP-seq via CUT&TAG

Chromatin immunoprecipitation quantitative PCR (ChIP-qPCR) and sequence (ChIP-seq) for tea plant was performed following previous researches [18, 35] with modification. Briefly, 2 g 0.5 cm^2^ pieces of spring-harvested tender leaves were cross-linked with 1% (v/v) formaldehyde under vacuum for 10 min. After grounding to powder in liquid nitrogen, the chromatin complexes were isolated and immunoprecipitated with Hyperactive pG-MNase CUT&RUN Assay Kit (Vazyme, Nanjing, China) and polyclonal antibodies to obtain CsBES1.2-binding DNA and input DNA. For the preparation of polyclonal antibodies, the peptide chain of 176-191aa at the C-terminal of CsBES1.2 (RGSKRKADWESFSNGS) was self-synthesized as antigen. Then, the polyclonal antibodies were purified from rabbit by ABclonal Biotechology Co. (Wuhan, China). For ChIP-qPCR, primer pairs were used to analyse the ChIP DNA. Three biological replicates (each containing two technical replicates) of fold enrichment on target gene promoters were calculated against *CsGAPDH*. Used primers in this experiment are listed in Table S8 (see online supplementary material).

The binding DNA and input DNA were then used for ChIP-seq according to previous researches [35]. In brief, pair-end sequencing of the sample was performed on the Illumina platform (Illumina, CA, USA) after library construction via Novogene Corporation (Beijing, China). Filtered raw sequencing reads were mapped to the tea plant genome (‘Shuchazao’ V2) by BWA mem (v 0.7.12). MACS2 (version 2.1.0) peak calling software was used to estimate fragment size and then detect the peak for IP enrichment regions. Finally, peak-associated genomic characteristics were annotated by using the R package ChIPseeker and mapped reads visualizations were performed by using IGV softerware.

### Electrophoretic mobility shift assay (EMSA)

pMAL-c5x-CsBES1.2 were transformed into *Transetta* (DE3) chemically competent cells. After confirming in SDS-PAGE, CsBES1.2-MBP in *Escherichia coli* cells were expressed with 0.5 mM IPTG at 37°C and purified with amylose resin. Oligonucleotide probes were synthesized and labeled with biotin at the 5′ end. Bases of candidate binding site were replace with poly adenine. The DNA binding activity of CsBES1.2 protein was detected by a Chemiluminescent EMSA Kit (Beyotime, Jiangsu, China). The reactions were analysed by electrophoresis in 6.5% polyacrylamide gels at 60 V for 65 min and were then transferred to a nylon membrane at 80 V for 60 min. Used primers in this experiment are listed in Table S8 (see online supplementary material).

### Yeast one-hybrid

Yeast one-hybrid assay was performed as former researches described [36]. The prey vector of pGADT7 was generated with the coding region of CsBES1.2. Two promoter fragments of CsEXL3, proP1 (1050 bp upstream of predicted translation start site and containing CGACGA as BRRE-box) and proP2 (785 bp upstream of predicted translation start site and containing CACGTG as E-box, named as *CsEXL3proP2*-pAbA) were amplified and inserted into pAbAi vector, respectively. Used primers in this experiment are listed in Table S8 (see online supplementary material).

## Supplementary Material

Web_Material_uhae074

## Data Availability

The accession numbers for the key genes described in this report are as follows: CsEXL3 (CSS0040385), CsBES1.2 (CSS0038858). The data for RNA-seq and ChIP-seq can be found in NCBI with the accession number PRJNA1075292. The genome data of 146 tea accessions for GWAS (Table S7, see online supplementary material) can be downloaded in TeaGVD (http://www.teaplant.top/teagvd) [30].
