# Supplementary material for: CsEXL3 regulate mechanical harvest-related droopy leaves under the transcriptional activation of CsBES1.2 in tea plant
Source: Hortic Res. 2024 Mar 7;11(5):uhae074. doi: 10.1093/hr/uhae074 (PMC11088715; doi:10.1093/hr/uhae074)
Supplement: Web_Material_uhae074 [file web_material_uhae074.zip › 231204 supplementary material forCsBES1.2and CsEXL3.docx]

Supplementary Material


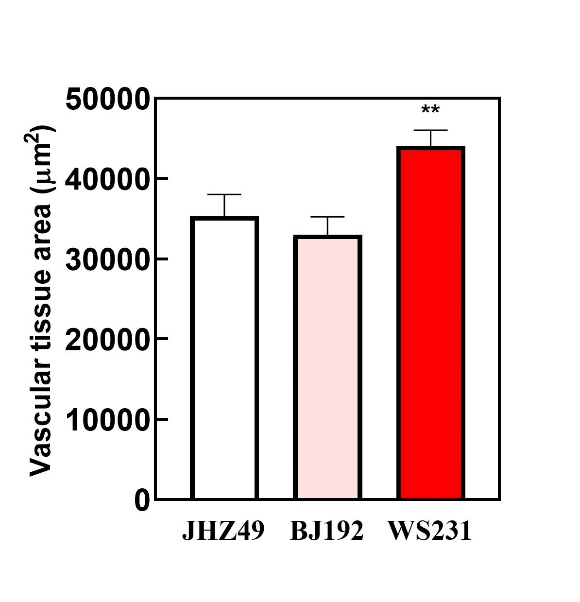


Figure S1. The vascular tissue area in the leaf blades midrib of selected germplasm, JHZ49, BJ192 and WS231.


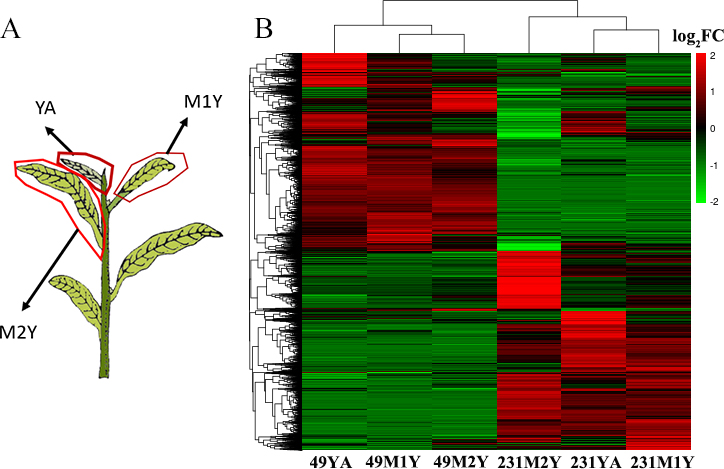


Figure S2. The heatmap and hierarchical clustering tree of different RNA-seq sets from the apical bud and 1st leaf (YA), first mature leaves (M1Y) and second mature leaves (M2Y) of JHZ49 and WS231. (A) The sample location of YA, M1Y and M2Y. (B) The hierarchical clustering tree for samples and genes of different RNA-seq sets.


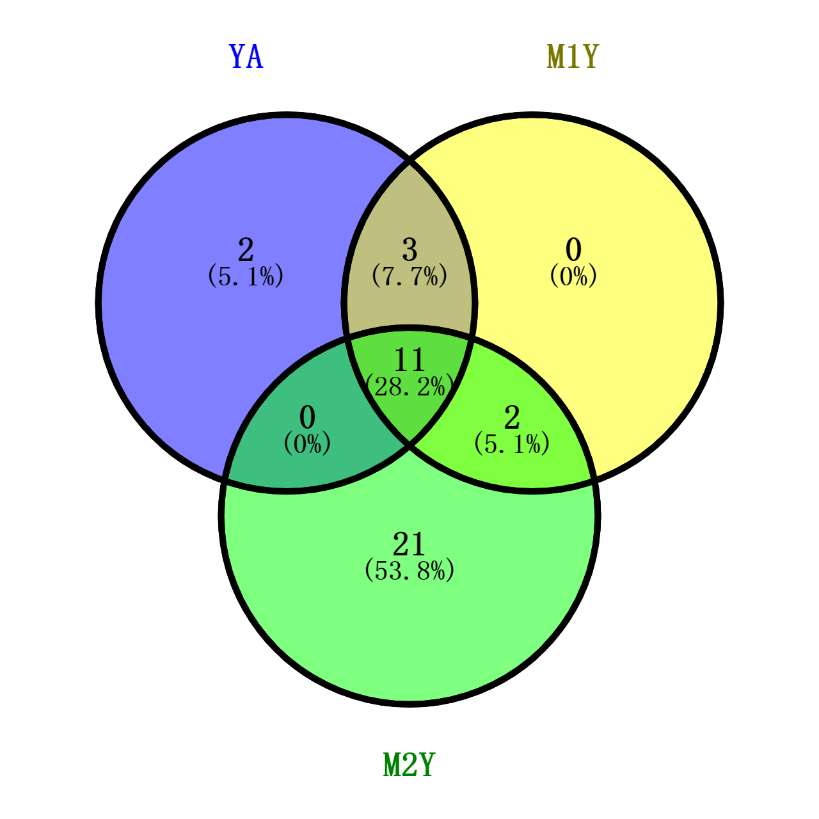


Figure S3. Venn diagram showing the number of common GO pathways in three type tissue of compared germplasms, JHZ49 and WS231. YA, the apical bud and 1st leaf; M1Y, first mature leaves; M2Y, second mature leaves.


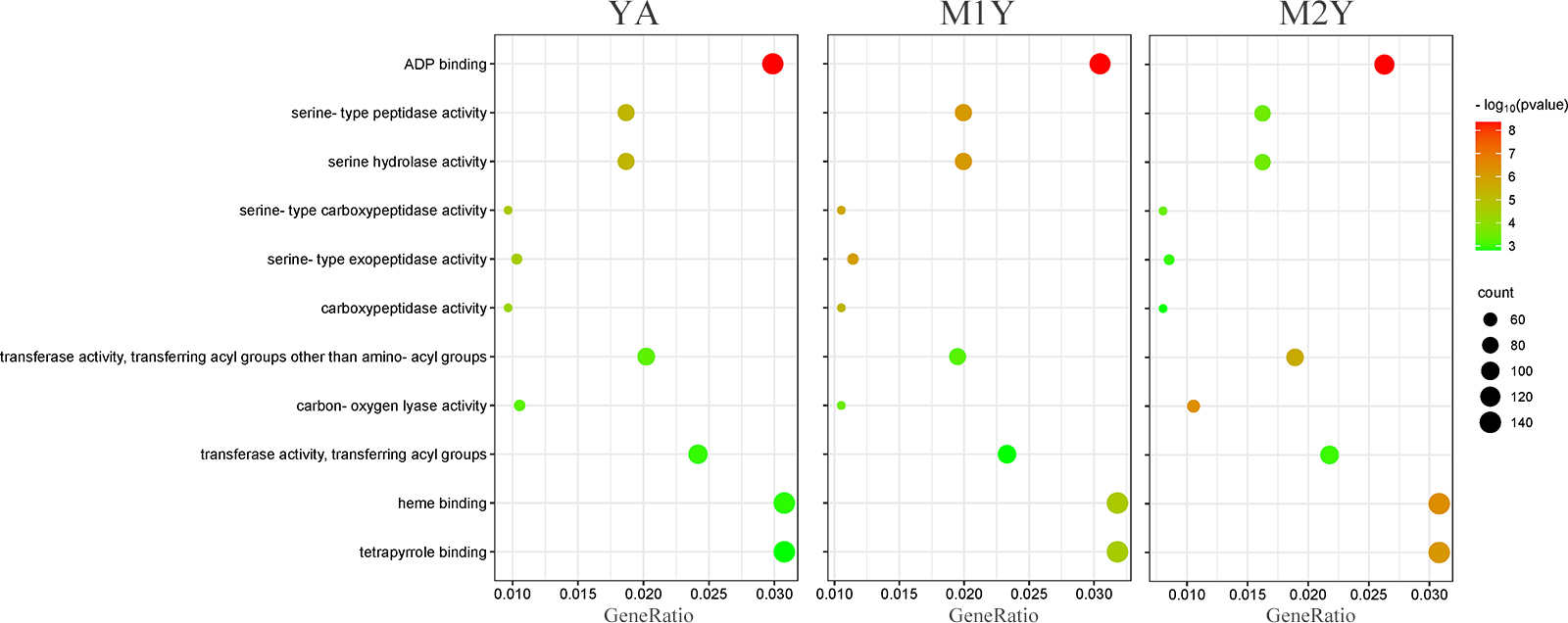


Figure S4. GO analysis of different RNA-seq sets from the apical bud and 1st leaf (YA), first mature leaves (M1Y) and second mature leaves (M2Y) of JHZ49 and WS231. Common significant GO pathways in YA, M1Y and M2Y were selected and shown when WS231 compared to JHZ49 (WS231/JHZ49).


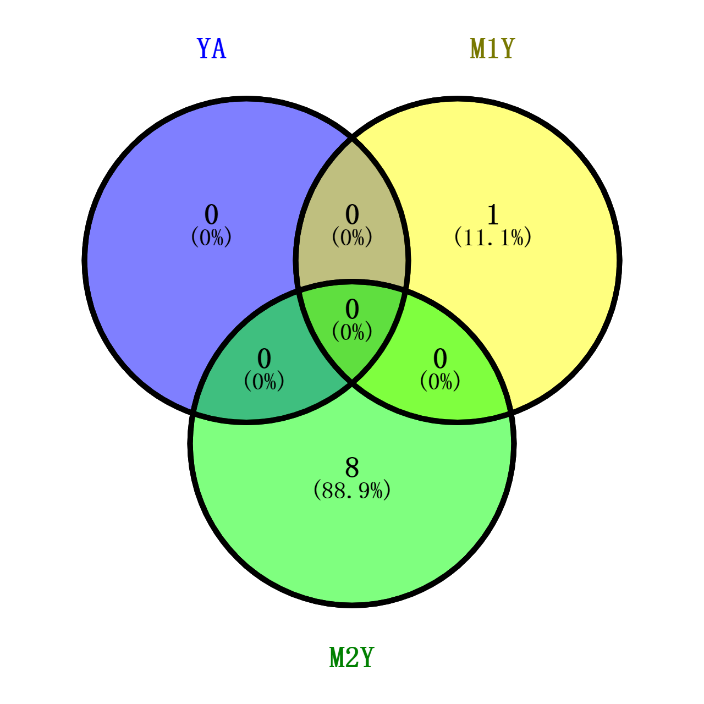


Figure S5. Venn diagram showing the number of common KEGG pathways in three type tissue of compared germplasms, JHZ49 and WS231. YA, the apical bud and 1st leaf; M1Y, first mature leaves; M2Y, second mature leaves.


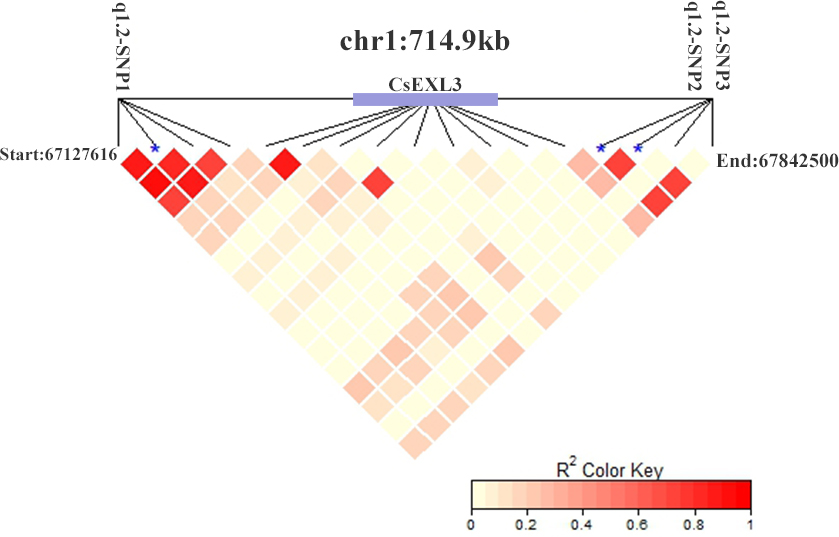


Figure S6. LD plot of 17 SNPs, including three significant association signals (q1.2SNP1, q1.2SNP2, q1.2SNP3), in a 0.71Mb (67127616–67842500 bp) region of chromosome 1. Asterisk indicated the location of q1.2SNP1, q1.2SNP2, and q1.2SNP3. dark purple box indicated the exon location of CsEXL3. CsEXL3 has no intron on the genome.


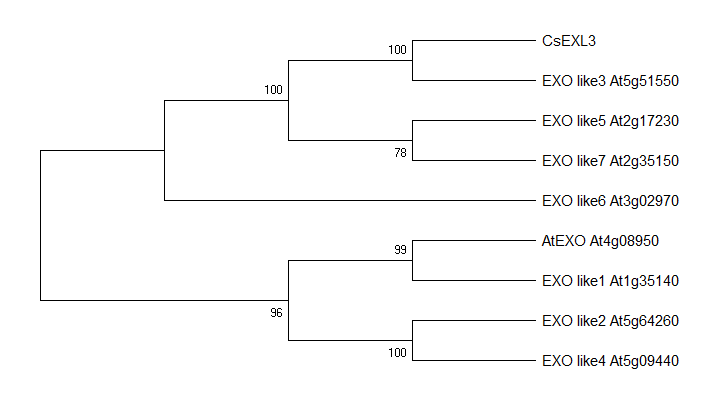


Figure S7. Phylogenetic tree of CsEXL3 protein from tea plant and EXO proteins from Arabidopsis. The phylogenetic tree is constructed based on the complete protein sequence alignment by the Neighbor-Joining method with bootstrapping analysis (1000 replicates) using MEGA X software.


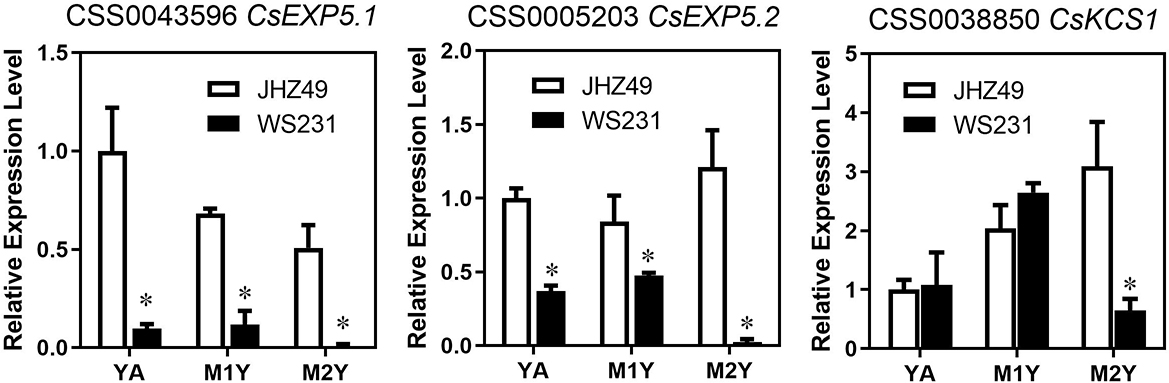


Figure S8. The relative expression levels of candidate *CsEXL3*-responsive genes, including *CsEXP5.1*, *CsEXP5.1*, and *CsKCS1*, in the apical bud and 1st leaf (YA), first mature leaves (M1Y) and second mature leaves (M2Y). Data presented are mean values (± standard error) of three biological replicates (n = 3), each consisting of a pooled sample of three fruit. Values with asterisk indicate significant difference at *p* < 0.05 while comparing to control group, JHZ49.


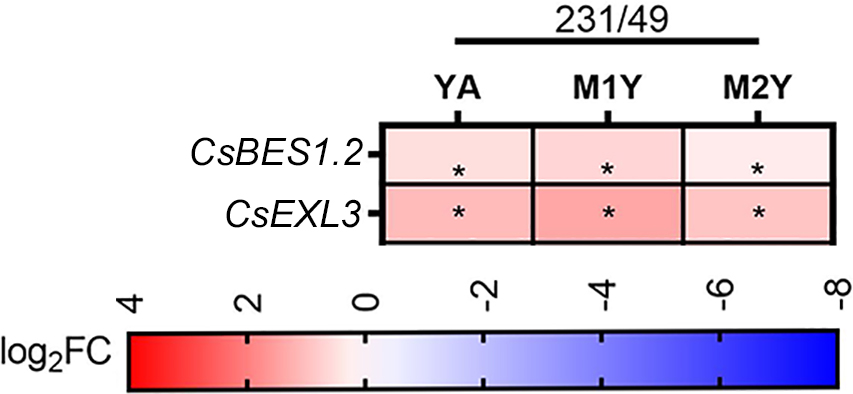


Figure S9. The heatmap of *CsBES1.2* and *CsEXL3* from three different type of leaves in JHZ49 (49) and WS231 (231). YA: the apical bud and 1st leaf, M1Y: first mature leaves, M2Y: second mature leaves. Values with asterisk indicate significant difference at *p* < 0.05 while comparing to control group, JHZ49.


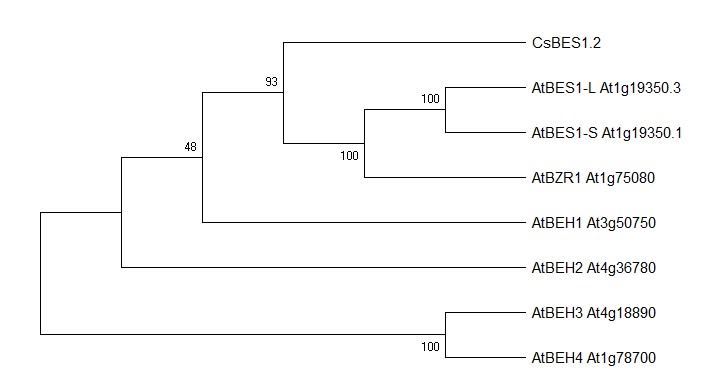


Figure S10. Phylogenetic tree of CsBES1.2 protein from tea plant and BES1 proteins from Arabidopsis. The phylogenetic tree is constructed based on the complete protein sequence alignment by the Neighbor-Joining method with bootstrapping analysis (1000 replicates) using MEGA X software.


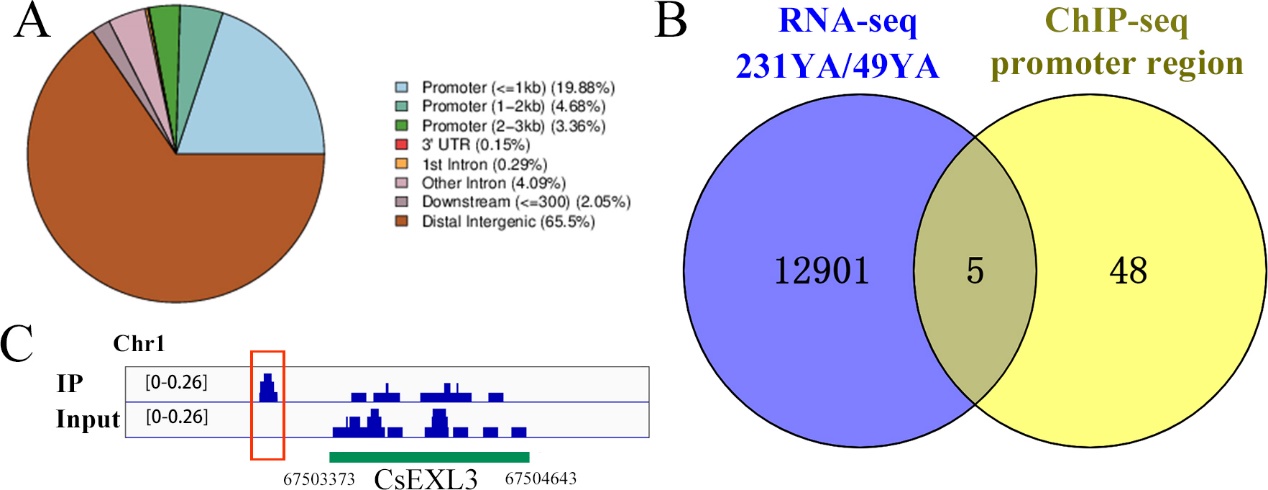


Figure S11. Genome-wide identification of CsBES1.2 directly regulated genes. (A) Genome-wide distribution analysis of CsBES1.2 binding peaks. (B) Venn diagram depicting number of CsBES1.2 target genes (53 from ChIP-seq) and DEGs in YA of WS231 when compared to JHZ49. (C) A screen shot of the ChIP-seq profile of CsBES1.2 at the CsEXL3 gene loci in WS231. The red line indicated the binding site of CsBES1.2 on promoter of CsEXL3.


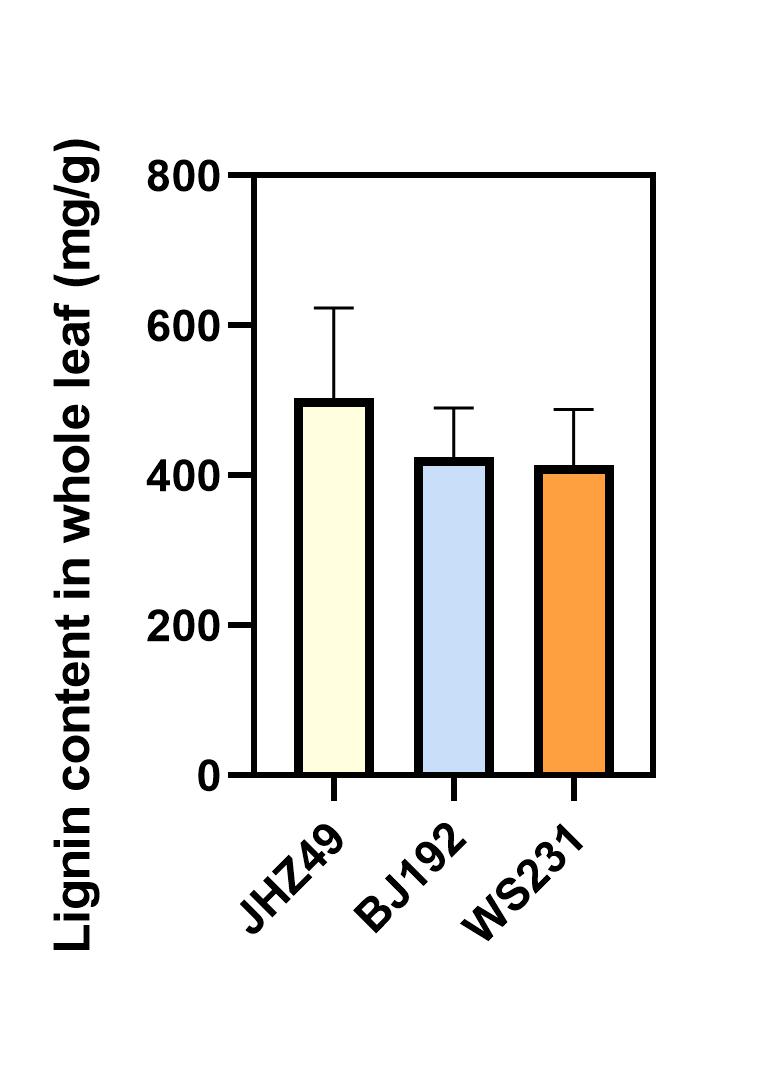


Figure S12. The lignin contents in the whole droopy or straight leaves of tea plant cultivars.


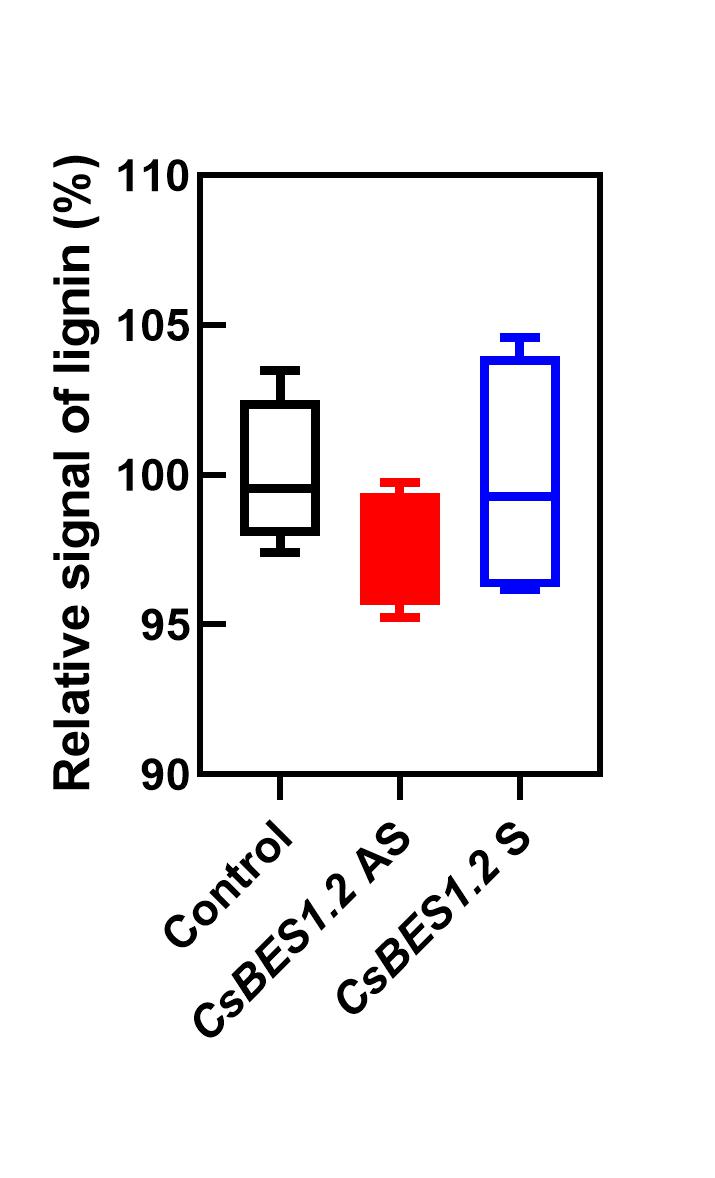


Figure S13. Relative lignin signals in *CsBES1.2*-silencing tea plants.
